# De novo transcriptomic analysis of leaf and fruit tissue of *Cornus officinalis* using Illumina platform

**DOI:** 10.1371/journal.pone.0192610

**Published:** 2018-02-16

**Authors:** Dian-Yun Hou, Lin-Chun Shi, Meng-Meng Yang, Jiong Li, Shuang Zhou, Hong-Xiao Zhang, Hua-Wei Xu

**Affiliations:** 1 Agricultural College, Henan University of Science and Technology, Luoyang, Henan Province, China; 2 The Luoyang Engineering Research Center of Breeding and Utilization of Dao-di Herbs, Luoyang, Henan Province, China; 3 Institute of Medicinal Plant Development (IMPLAD), Chinese Academy of Medical Sciences & Peking Union Medical College, Beijing, China; 4 Chinese Medicinal Materials Production Technology Service Center, Department of Agriculture of Henan Province, Zhengzhou, Henan Province, China; Huazhong University of Science and Technology, CHINA

## Abstract

*Cornus officinalis* is one of the most widely used medicinal plants in China and other East Asian countries to cure diseases such as liver, kidney, cardiovascular diseases and frequent urination for thousands of years. It is a Level 3 protected species, and is one of the 42 national key protected wild species of animals and plants in China. However, the genetics and molecular biology of *C*. *officinalis* are poorly understood, which has hindered research on the molecular mechanism of its metabolism and utilization. Hence, enriching its genomic data and information is very important. In recent years, the fast-growing technology of next generation sequencing has provided an effective path to gain genomic information from nonmodel species. This study is the first to explore the leaf and fruit tissue transcriptome of *C*. *officinalis* using the Illumina HiSeq 4000 platform. A total of 57,954,134 and 60,971,652 clean reads from leaf and fruit were acquired, respectively (GenBank number SRP115440). The pooled reads from all two libraries were assembled into 56,392 unigenes with an average length 856 bp. Among these, 41,146 unigenes matched with sequences in the NCBI nonredundant protein database. The Gene Ontology database assigned 24,336 unigenes with biological process (83.26%), cellular components (53.58%), and molecular function (83.93%). In addition, 10,808 unigenes were assigned a KOG functional classification by the KOG database. Searching against the KEGG pathway database indicated that 18,435 unigenes were mapped to 371 KEGG pathways. Moreover, the edgeR database identified 4,585 significant differentially expressed genes (DEGs), of which 1,392 were up-regulated and 3,193 were down-regulated in fruit tissue compared with leaf tissue. Finally, we explored 581 transcription factors with 50 transcription factor gene families. Most DEGs and transcription factors were related to terpene biosynthesis and secondary metabolic regulation. This study not only represented the first de novo transcriptomic analysis of *C*. *officinalis* but also provided fundamental information on its genes and biosynthetic pathway. These findings will help us explore the molecular metabolism mechanism of terpene biosynthesis in *C*. *officinalis*.

## Introduction

*C*. *officinalis* Siebold et Zucc. (Cornaceae) is a deciduous tree widely distributed in China, Korea and Japan [1, 2]. The dried ripe and stoneless fruit of *C*. *officinalis* is a well-known Traditional Chinese Medicine (TCM), known as “Corni Fructus” or “Shan Zhu Yu” in China [3] and is mainly produced in the Henan, Shanxi, and Sichuan provinces etc [4, 5]. *C*. *officinalis* is classified as a food and medicinal plant by the Ministry of Health of the People’s Re- public of China. It is the main ingredient of a medicine named Liu wei di huang pill. Corni Fructus has become a famous medicinal plant because of its variety of bioactivities, such as anti-inflammatory, anti-diabetes, anti-tumor, anti-oxidant, and anti-aging activities [6–10]. Corni Fructus has been used to treat various diseases, including vertigo, tinnitus, cardiovascular disease, frequent urination, oxidative stress, and acute myocardial ischemia [3, 5, 11]. Moreover, it also can regulate the immune system [12–14]. Studies have increasingly discovered the natural constituents present in *C*. *officinalis* such as secoiridoid glycosides [1,15], bisiridoid glycosides [16], iridoid glucosides [1,10] and ursolic acid [17]. The previous results show that iridoid glucosides are its main active substance [3, 5, 10].

*C*. *officinalis* is an important medicinal plant with a number of pharmacological uses. Although significant progress has been made to determine its physiological function, chemical composition, and pharmacologically active components, studies on the biochemical and molecular mechanisms of iridoid glucoside biosynthesis, involving transcriptome and gene expression analysis in *C*. *officinalis* are rare. Until 15 July 2017, there are 429 microsatellite loci sequences and 26 protein sequences of C. *officinalis* deposited in the NCBI GenBank data-base. The shortage of transcriptomic information and gene database has been the bottleneck for further studies on the biosynthetic mechanisms of iridoid glucosides in *C*. *officinalis*. Thus, it is imperative to obtain its transcriptome data.

RNA sequencing (RNA-seq) is revolutionizing the study of the transcriptome. It is a highly sensitive and accurate tool for measuring expression across the transcriptome [18, 19]. Recently, the advent and decreasing cost of next generation sequencing technology make RNA-seq a more effective choice for transcriptomics studies [20, 21]. Nowadays, RNA-seq has been widely deployed to reveal the biosynthetic pathways in the secondary metabolism of many medicinal plants, such as *Cannabis sativa* [22], *Panax ginseng* [23, 24], *Glycyrrhiza uralensis* [25], *Calotropis procera* [26] and *Mentha species* [27]. Currently, RNA-seq has not been utilized to study *C*. *officinalis*.

In this study, we used the Illumina transcriptome sequencing technology to sequence a transcriptome library generated from leaf and fruit of *C*. *officinalis*. The results will help us to understand the gene expression difference and explore the molecular mechanisms of biosynthetic pathways in secondary metabolites in *C*. *officinalis*.

## Materials and methods

### Plant material collection

*C*. *officinalis* is perennial plant, the fruits of which matures around October to November every year in different areas of distribution and is characterized by the development of red color. In this study, samples of mature *C*. *officinalis* fruits and leaves were collected at the Luoyang of Henan Province on 20 October 2016. All samples were immediately frozen in liquid nitrogen and stored at -80°C until RNA extraction.

### RNA extraction and cDNA library preparation

Total RNA from approximately 80 mg of frozen tissue of leaves (coYP) and fruits (coGS) was extracted using the TRIzol^®^ Reagent (Invitrogen, USA) according to manufacturer’s protocol. RNA quality was assessed by NanoDrop^™^2000 spectrophotometer (NanoDrop Technologies, USA). All RNA extracts showed a 260/280 nm ratio of 1.8 to 2.2. Approximately 1 μg total RNA of no less than 50 ng/μL concentration, was used for RNA-seq library construction. The cDNA library was constructed using Truseq^™^ RNA sample preparation Kit (Illumina, San Diego, CA, USA) following the manufacturer’s protocol. Poly-(A) mRNA was isolated from total RNA through Oligo-(dT) magnetic beads, and then fragmented in fragmentation buffer. The mRNA was randomly cut into 200 bp segments. The first strand cDNA was synthesized using short fragments, whereas the second strand cDNA was synthesized by second strand synthesis mix. The cDNA was repaired with End Repair Mix and addition of “A” base adaptors were ligated to the cDNA molecules.

### RNA Illumina Hiseq sequencing

The cDNA library was enriched using 15 cycles of PCR. Subsequently, the target band was recycled using 2% low range ultra-agarose, and quantified by TBS 380 Mini-Fluorometer. The suitable libraries were sequenced using Illumina HiSeq 4000 SBS Kit (300 cycles, Illumina, San Diego, CA, USA) at Shanghai Majorbio Bio-pharm Biotechnology Co., Ltd. (Shanghai, China). The raw sequence reads from the Illumina sequencing were deposited in the NCBI Sequence Read Archive (SRA).

### De novo assembly

First, raw reads with adaptor were trimmed, vector contaminated and low quality reads (base *Q value* < 20) were discarded. Second, whole reads with a base *Q value* < 10 were discarded. Lastly, reads with “N” bases and lengths below 20 were removed. All the above mentioned processes were carried out using the SeqPrep (https://github.com/jstjohn/SeqPrep) and Sickle(https://github.com/najoshi/sickle) [28]. Because of the absence of a reference genome, the high quality reads of leaves and fruits of *C*. *officinalis* were used for de novo assembly using the Trinity (http://trinityrnaseq.sourceforge.net/) [28].

### Functional annotation and classification of unigenes

The unigenes obtained in the transcriptome were aligned using NCBI BlastX function (E value<10^−5^) against the non-redundant (NR) sequence database, Swissprot (http://www.expasy.ch/sprot), KEGG (Kyoto Encyclopedia of Genes and Genomes, http://www.genome.jp/kegg/), KOG (Clusters of Orthologous Groups for Eukaryotic Complete Genomes, http://www.ncbi.nlm.nih.gov/KOG/), and GO (Gene Ontology, http://www.geneontology.org) [29–32]. GO function classification was performed using Blast2GO (http://www.blast2go.com/b2ghome), which allowed categorization into three different three GO terms, including Biological Process, Molecular Function and Cellular Component [33]. KEGG (Kyoto Encyclopedia of Genes and genomes) was used to determine pathway through Blastll software against the KEGG database [34].

### Expression analysis

After assembly, high quality RNA-seq reads were used to determine alignment counts and quantify transcript abundance using the RSEM package (RNA-seq by Expectation Maximizattion, http://www.biomedsearch.com/nih/RSEM-accurate-transcript-quantification-from/21816040.html) using minimum and maximum fragment lengths of 200 and 300bp, respectively [35]. RSEM can be used to calculate the number of RNA reads or fragments mapped to unigenes based on FPKM (fragments Per Kilobase per Million) values [36].

DEGs between leaves and fruit tissue of *C*. *officinalis* were identified using the R Bioconductor package edgeR (http://www.bioconductor.org/packages/2.12/bioc/html/edgeR.html) was used [37]. The FDR (false discovery rate) is used to determine p-valuethresholds in multiple testing [38, 39]. The significance of DEGs were determined based on a threshold of FDR<0.05 and absolute value of log_2_fold change ≥1 (log_2_fc ≥1). DEGs were subsequently mapped to the database for pathway enrichment analysis [21].

In addition, the GO and KEGG enrichment analysis of DEGs were approved using the software GOatools (https://github.com/tanghaibao/GOatools) [40, 41] and KOBAS (http://kobas.cbi.pku.edu.cn/home.do) [42] with the p-value< 0.05.

### Transcription factor analysis

The transcription factors in the transcriptome of *C*. *officinalis* leaf and fruit tissues were predicted by searching against Plant TFDB 3.0 (http://planttfdb.cbi.pku.edu.cn/) using BlastP with the E-value cut off set to1e-^8^ (E-value <1e-8) [43].

## Results

### Illumina sequencing and de novo assembly

The cDNA library was constructed from the fruits and leaves of *C*. *officinalis* using TruseqTM RNA sample preparation kit. Subsequently, the high-quality library was sequenced using the Illumina transcriptome platform. Since the genome of *C*. *officinalis* is not available, we performed de novo assembly of the transcripts. We obtained 62,026,372 and 59,147,826 raw reads for the fruit (coGS) and leaf (coYP), respectively, which consist of 9,365,982,172 bp and 8,931,321,726 bp. For the quality control of raw reads, we have calculated the composition (S1A and S1B Fig), quality (S1C and S1D Fig), and error rates (S1E and S1F Fig) of A/T /C/G bases in the raw reads. Results showed that the reads meet the quality needed for further analysis. After eliminating random primer and adapter sequences and removing low-quality reads and short sequences of less than 20 bp, we obtained 60,971,652 and 57,954,134 clean reads of fruit coGS and coYP, which contained 9,044,801,270 and 8,579,879,557 bp, respecttively. In addition, the Q20% and Q30% were 98.08 and 97.94 for fruit and 93.97 and 93.58 for leaf, respectively. Moreover, the GC content of coGS and coYP was 45.98% and 46.54%, respectively. The sequences can be found in NCBI SRA with the accession number SRP 115440. For the assembly, a total of 56,392 unigenes consisting of 48,264,743 bp have been acquired. The percentage GC of all the unigenes was 43.74%, and the average length of each unigene was 856 bp. The largest and smallest unigenes were 124,880 and 201 bp in length, respectively. The N50 was 1445 bp (Table 1).

**Table 1:** Assembly results of unigenes and transcripts from fruit and leaf tissue of *C*. *officinalis*.

Type	Unigene	Transcripts
Total sequence num	56,392	70,329
Total sequence base (bp)	48,264,743	66,767,256
Percent GC (%)	43.74	43.34
Largest (bp)	124,880	124,880
Smallest (bp)	201	201
Average (bp)	856	946
N50 (bp)	1445	1536
N90 (bp)	302	366

### Functional annotation of unigenes

**Fig 1:**
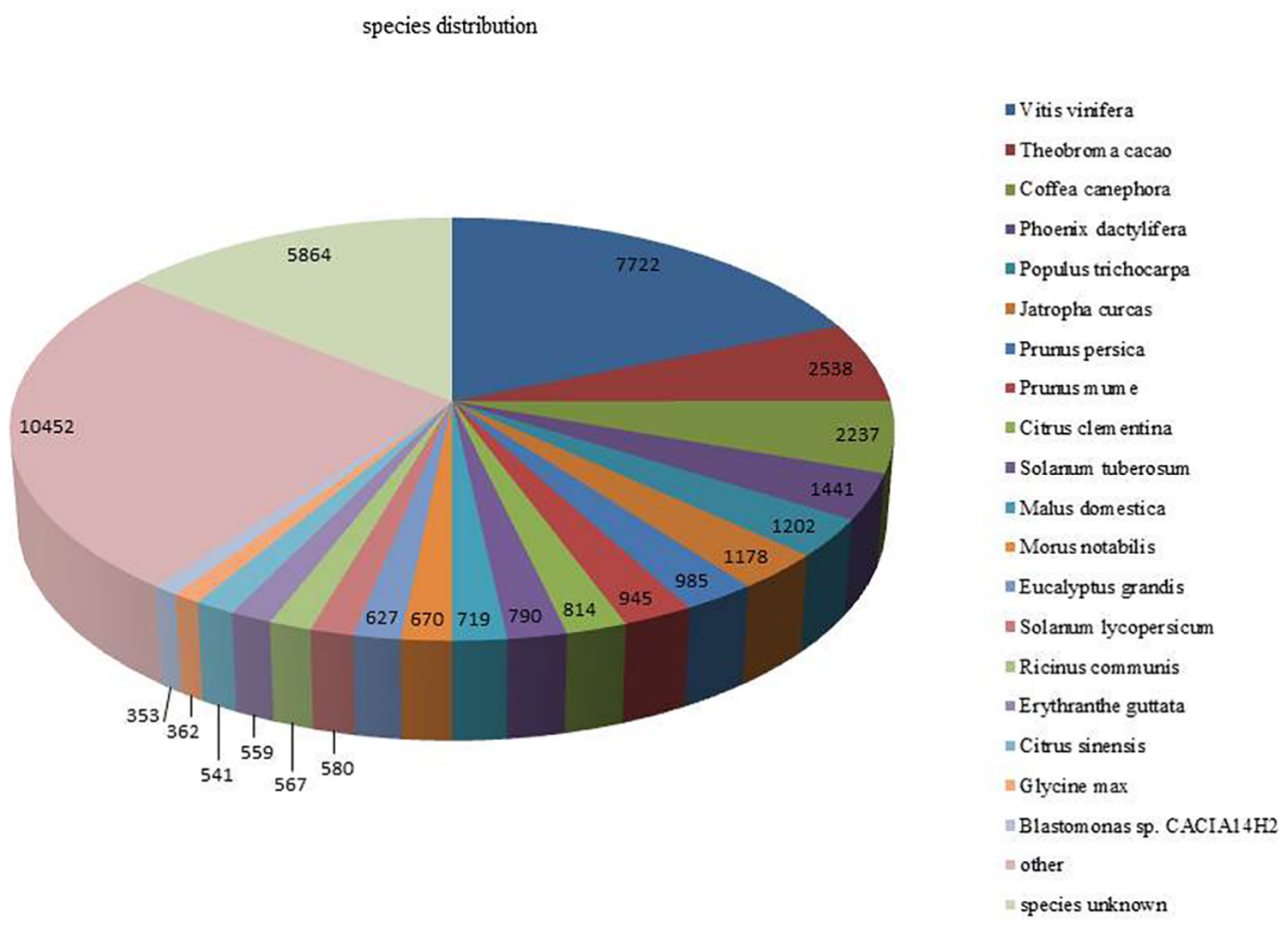
Results of species distribution of sequence homology search against NCBI NR database for *C*. *officinalis* unigenes.

For functional annotation, the unigenes were searched against public databases NR, GO, and KEGG using BlastX, with an E-value cut-off set to 1x10^-5^. Different unigenes have been matched in different databases, for example, 41,146 unigenes were matched in NR database. Unigene sequences were searched against the NR database for annotation revealed 7,722 unigenes (18.77%) matched to *Vitis vinifera* and 2,538 unigenes (6.17%) matched to *Theobroma cacao*, as shown in Fig 1 and S1 Table.

### Functional classification of unigenes

**Fig 2:**
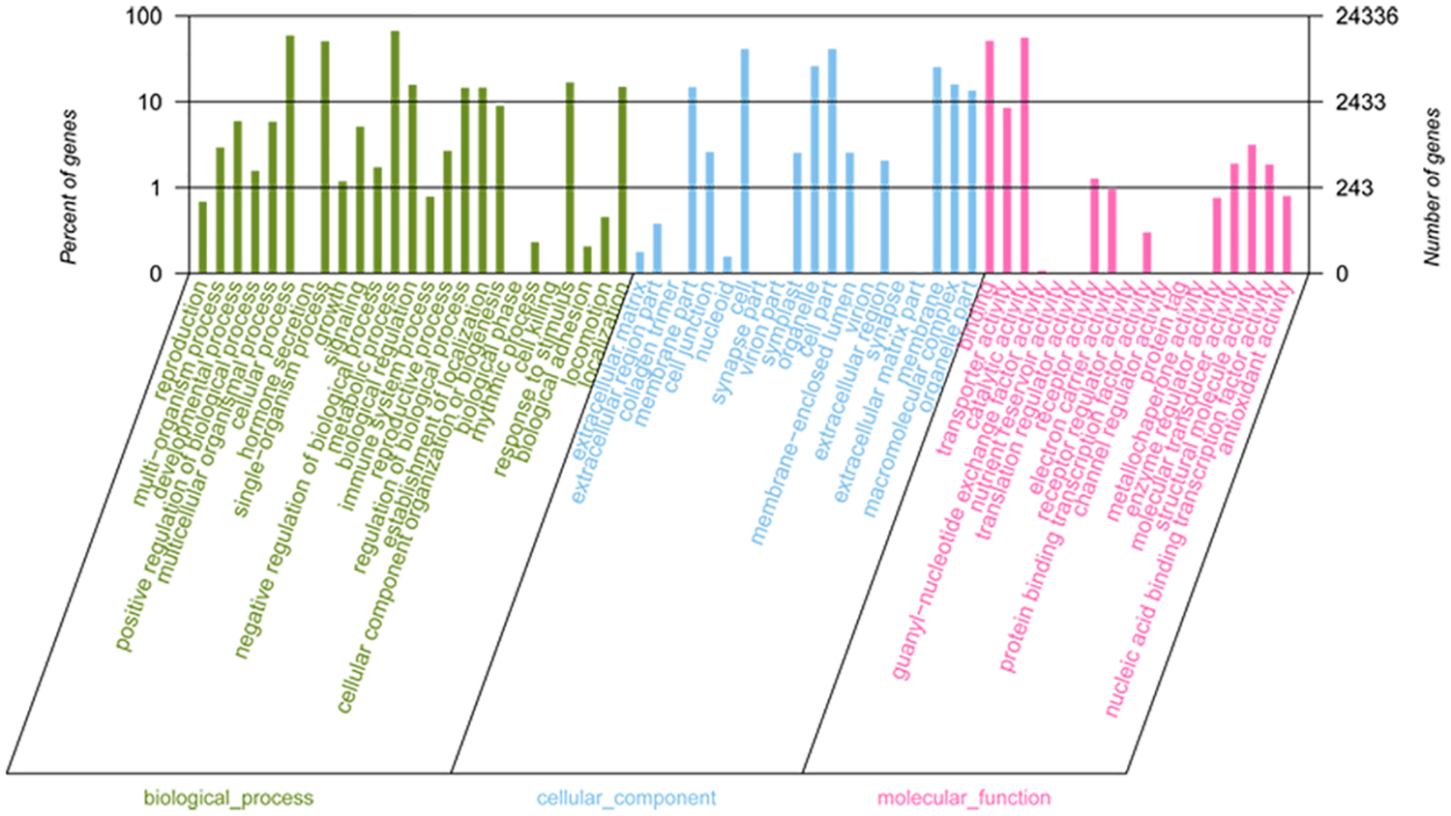
Gene Ontology (GO) term level2 categories of unigenes of *C*. *officinalis*.

Functional classification of unigenes by GO assignments resulted in the successful annotation of 24,336 unigenes. These unigenes were summarized into three main GO categories biological process (BP, 20263 unigene; 83.26%), cellular components (CC, 13040; 53.58%), and molecular function (MF, 20424; 83.93%) (Fig 2, S2 Table). Under the biological process category, metabolic process (16,201; 66.57%), cellular process (14,351; 58.97%), single organism process (12,297; 50.53%) and biological regulation (3,823; 15.71%) were the most dominant subcategories, as shown in Fig 2 and S2 Table. Furthermore, the cellular components category mainly consisted of cell (9,983; 41.02%), cell part (9,983; 41.02%), organelle (6,316; 25.95%), membrane part (3,268; 13.43%), macromolecular complex (3,874; 15.72%), and membrane (6,144; 25.25%). Lastly, the molecular function category mainly consisted of binding (12,461; 51.20%), catalytic activity (13,557; 55.71%), transporter activity (2,064; 8.48%), and structural molecular activity (764; 3.14%), as shown in Fig 2 and S2 Table.

**Fig 3:**
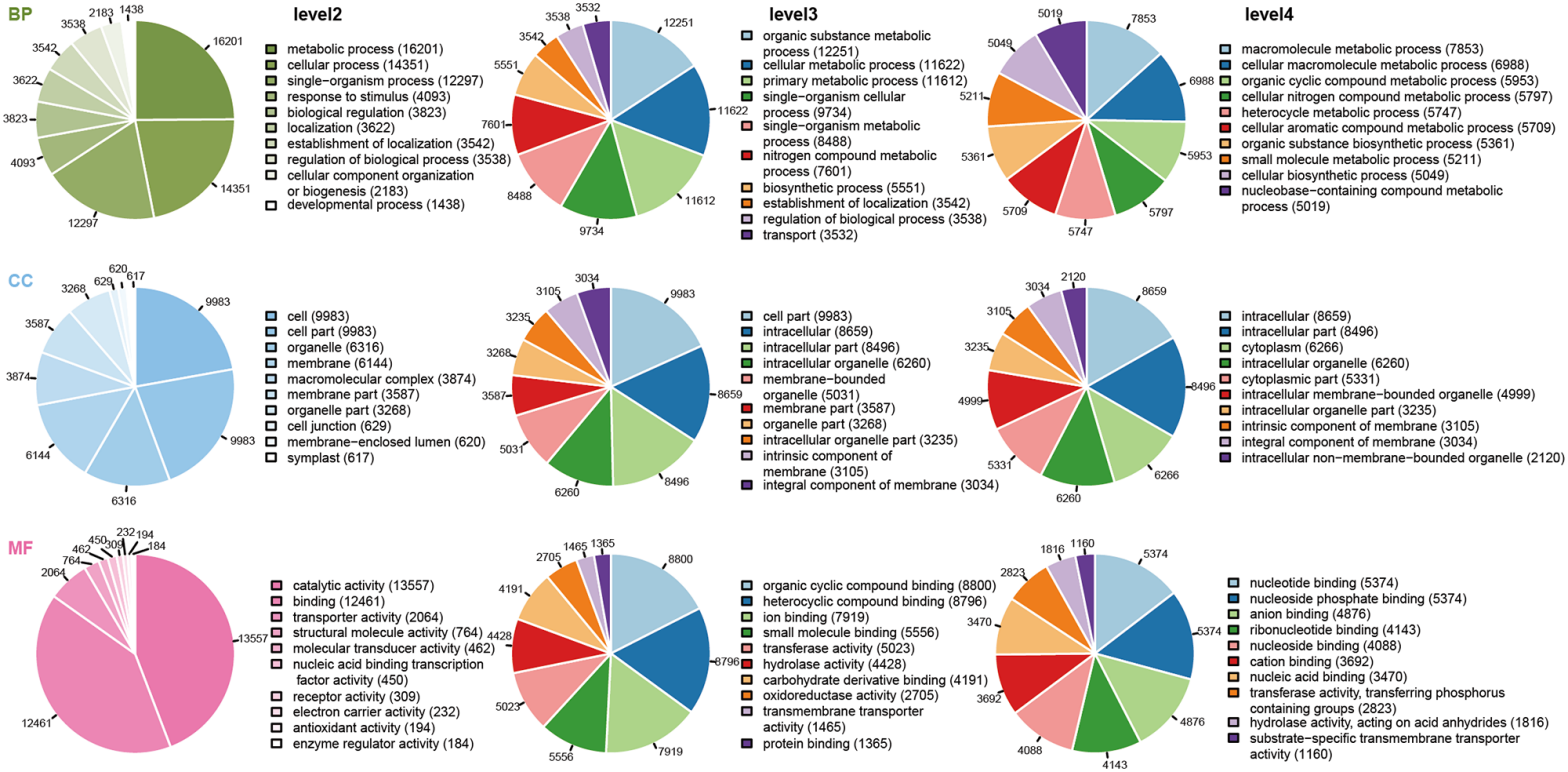
GO term level 2,3 categories of unigenes of *C*. *officinalis*.

For extensive analysis of function based on the GO database, each of the three categories were subcategorized into level 3 and level 4 terms for molecular function. For example, transporter activity is the main level 2 term for molecular function, and in level 3 terms, transporter activity was assigned to the following specific functions: substrate specific transporter activity (GO: 0022892; 1269 unigene; 5.21%), drug transporter activity (GO: 0090484; 82; 0.34%), cofactor transporter activity (GO: 0051184; 16; 0.07%), isomerase activity (GO: 0016853; 419; 1.72%) (Fig 3, S3 Table). Moreover, in level 4 terms, substrate specific trans-membrane transporter activity (belonging to transporter activity) is subcategorized to nicotinate transporter activity (GO: 0090416; 1; 0.004%), substrate specific transmembrane transporter activity (GO: 0022891; 1160; 4.767%), oxygen transporter activity (GO: 0005344; 5; 0.021%), lipid transporter activity (GO: 0005319; 40; 0.164%), protein transporter activity (GO: 0008565; 87; 0.3575%), N-methylnicotinate transporter activity (GO: 0090417; 1; 0.0041%), and peptide transporter activity (GO: 0015197; 24; 0.0986%) (Fig 3, S4 Table).

**Fig 4:**
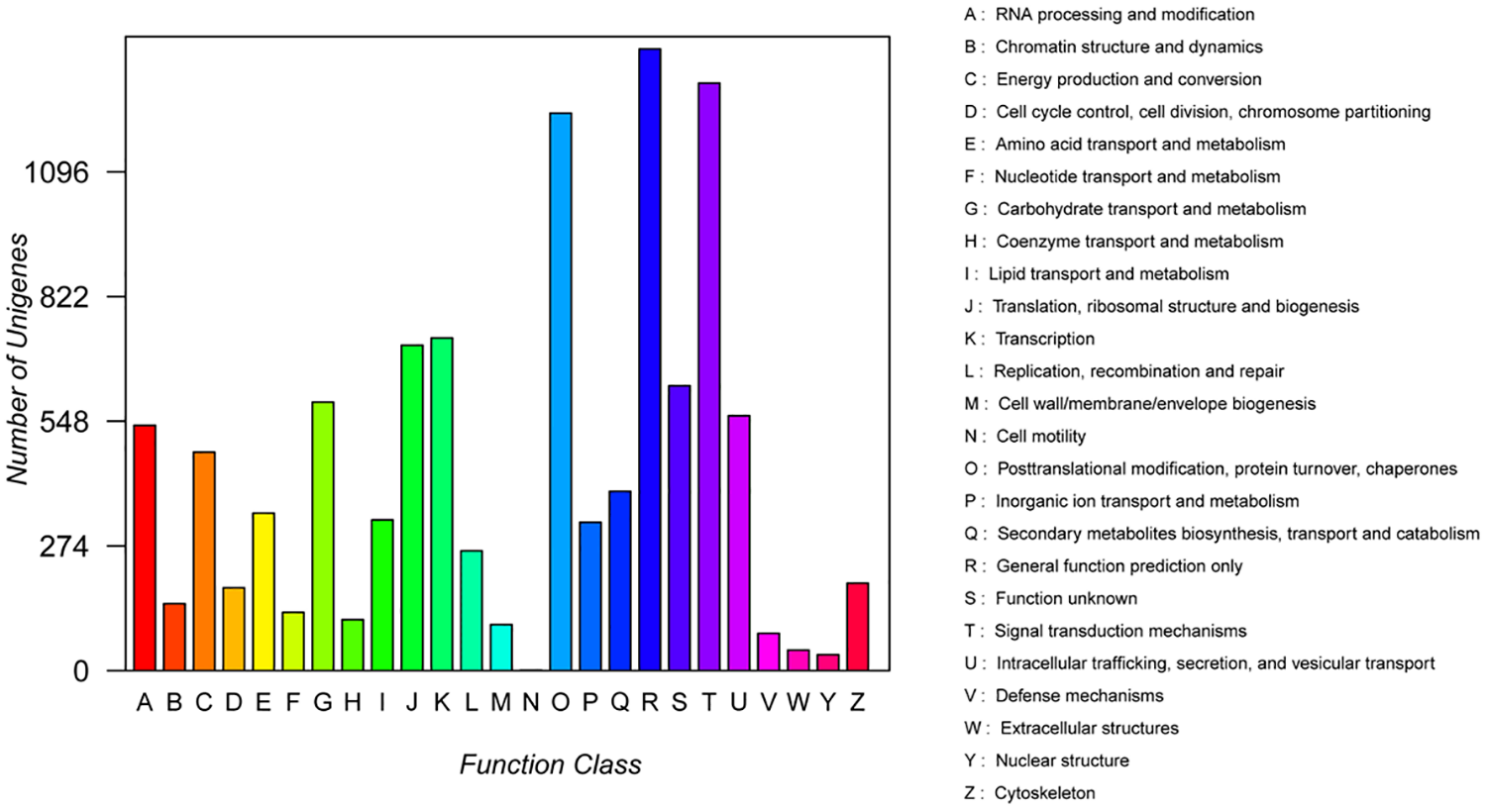
KOG functional classification of *C*. *officinalis* unigenes. A total of 10808 unigenes with significant homology to the KOG database (E-value≤1.0 E^-5^) were classified into 25 categories.

To further evaluate the annotated unigene data, the unigene sequences were searched against the KOG databases. The query returned 10,808 unigene sequences assigned to 25 KOG categories under the following types of information: storage and processing, cellular processes and signaling, metabolism, and poorly characterized (Fig 4, S5 Table). Among the 25 categories, which were alphabetized, “general function prediction only(R)” was the topmost category (1,366; 12.64%), followed by “signal transduction mechanisms (T)” (1291;11.94%), “posttranslational modification, protein turnover, chaperones (O)” (1,225; 11.33%),“transcription (K)” (731; 6.76%), “translation, ribosomal structure and biogenesis (J)”(715;6.62%), “carbohydrate transport and metabolism (G)” (590; 5.46%), and “amino acid transport and metabolism (E)” (346; 3.20%). However, because of the limited molecular information on *C*. *officinalis* and its related species on the KOG database, there are 626 unigenes (5.79%) with no indicated function (Fig 4, S5 Table). Similar results have been reported in other plant species [32, 39].

**Fig 5:**
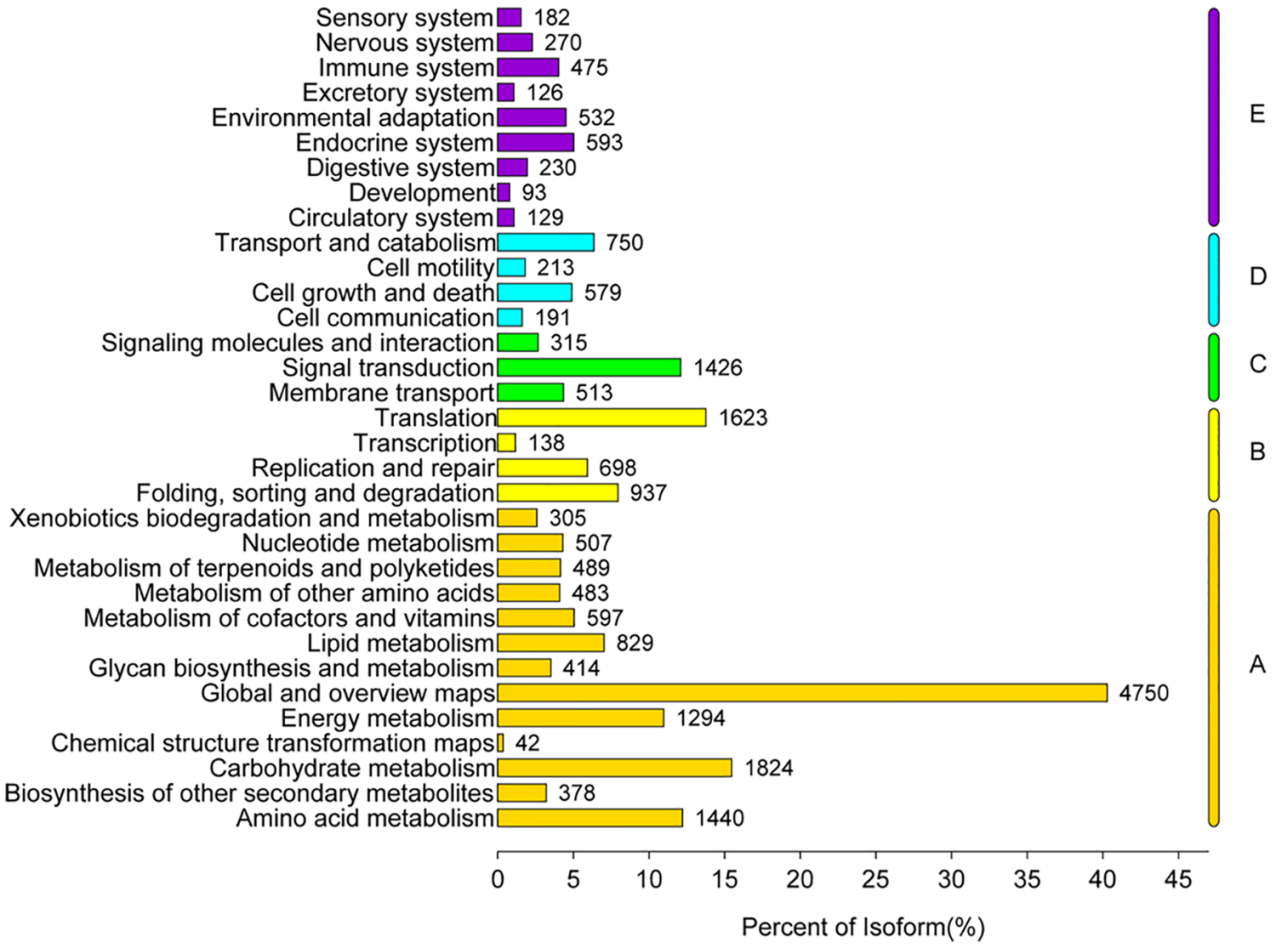
Classification map of KEGG metabolic pathway. A: Metabolism, B: Genetic information processing, C: Environmental information processing, D: Cellular processes, E: Organismal systems.

The KEGG database is used to identify the biochemical pathways assigned to unigene sequences. In our results, a total of 18,435 unigenes sequences were assigned to 371 KEGG path ways (S6 Table). The pathways with the highest unigene representations were those related with metabolic pathways (ko01100; 4,451 unigenes, 24.14%), biosynthesis of secondary metabolites (ko01110; 2,281, 12.38%), biosynthesis of antibiotics (ko01130; 1,330, 7.21%), microbial metabolism in diverse environments (ko01120; 1,245, 6.75%), carbon metabolism (ko01200; 870, 4.72%), and ribosome (ko03010; 685, 3.72%) (S6 Table) of the other assignment was presented in S6 Table. In addition, all the pathways could be divided into 5 branches, including metabolism (A), genetic information processing (B), environmental information processing (C), cellular processes (D), and organismal systems (E) (Fig 5, S7 Table), with each branch of pathway containing different sub-pathways. For example, cellular processes (D) are involved in transport and catabolism, cell growth and death, cell communication, and cell motility (Fig 5, S7 Table).

### Expression profiling of unigenes

To explore the differential expression of unigenes in coGS and coYP of *C*. *officinalis*, clean reads from every tissue library were mapped to our unigene database through the RSEM package. Results showed that 50,029,300 reads were aligned in fruits, which was 82.05% of original clean reads (60,971,652), and 48,063,854 reads were aligned in leaves, which was 82.93% of original clean reads (57,954,134). Using the RSEM software, the number of reads or fragments mapped to unigenes and the expression levels of unigenes in fruit and leaf were calculated based on FPKM method. S8 Table demonstrates that different unigenes could be supported by different numbers of reads in fruit and leaf. For example, 958 and 2373 reads were aligned in unigene c70915_g1in coGS and coYP, respectively (S8 Table) In addition, we also determined the FPKM expression quantity of every gene in coGS and coYP (S9 Table), as well as the FPKM expression density scatter gram in coGS and coYP (S2 Fig). Moreover, S2 Fig. indicates all the gene expression density distribution in coGS and coYP. Log2FPKM4-5 was the most concentrated area of gene expression quantity.

### Identification and analysis of differentially expressed genes (DEGs)

Using edgeR, we studied the DEGs in coGS and coYP based on FDR, which is a statistical method to test the correction for comparisons. To further explore characteristic up-regulated and down-regulated expressed genes in the coYP versus coGS, genes with significant differential expression were determined based on the threshold of FDR< 0.05, log2FC ≥ 1 and *p* value < 0.05. According to the standard, 4,585 significant DEGs were identified, with 1,392 being up-regulated and 3,193 being down-regulated (Table 2, S10 Table). When the significance of differential expression was not considered, 26,136 DEGs were obtained, with 11,735 being up-regulated and 14,401 being down-regulated genes (Table 2, S11 Table).

**Table 2:** Statistics of DEG of coYP versus coGS.

name	count
Total of unigenes/genes available	56392
No. of DEGs	26136
No. of up-regulated DEGs (coYP/coGS)	11735
No. of down-regulated DEGs	14402
No. of significant DEGs	4585
No. of significant up-regulated DEGs (coYP/coGS)	1392
No. of significant down-regulated DEGs	3193

### GO classified statistic and enrichment analysis of DEGs

**Fig 6:**
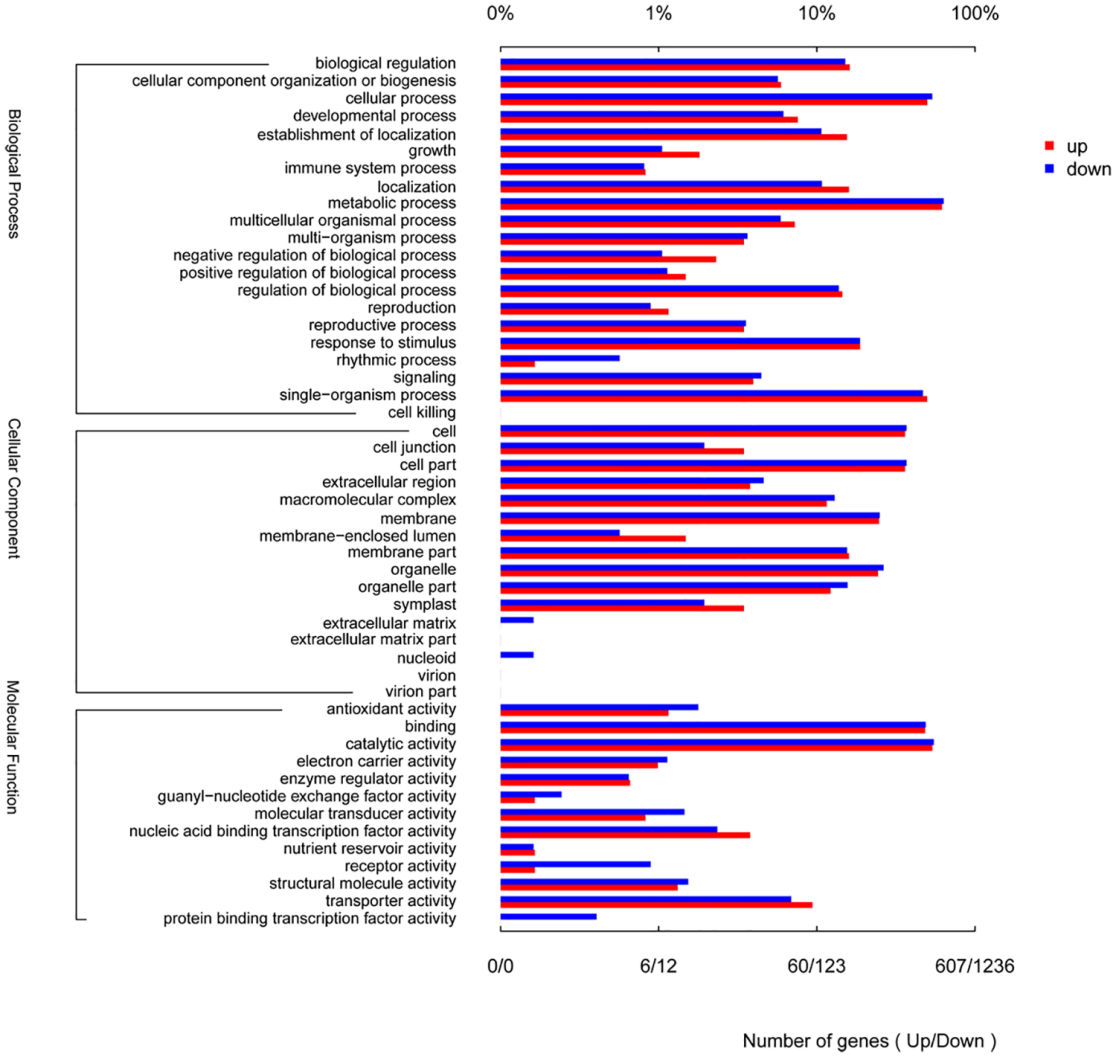
GO annotation of up- and down-regulated expression genes of coYP versus coGS.

Using the GO database, the significant DEGs were represented in the three main GO categories, which include biological process, cellular components, and molecular function (Fig 6, S12 Table). In the GO biological process category, with coYP as contrast, the gene expression of metabolic process was obvious. There were 375 up-regulated expression genes and 783 down-regulated expression genes. Subsequently, a cellular process with 303 up-regulated and 662 down-regulated expression genes ensued. In the GO molecular function category, with coYP as contrast, gene expression of catalytic activity was significant. There were 326 up-regulated expression genes and 679 down-regulated expression genes. Then, binding with 293 up-regulated and 603 down-regulated expression genes ensued. The detailed classification could be found in Fig 6 and S12 Table.

**Fig 7:**
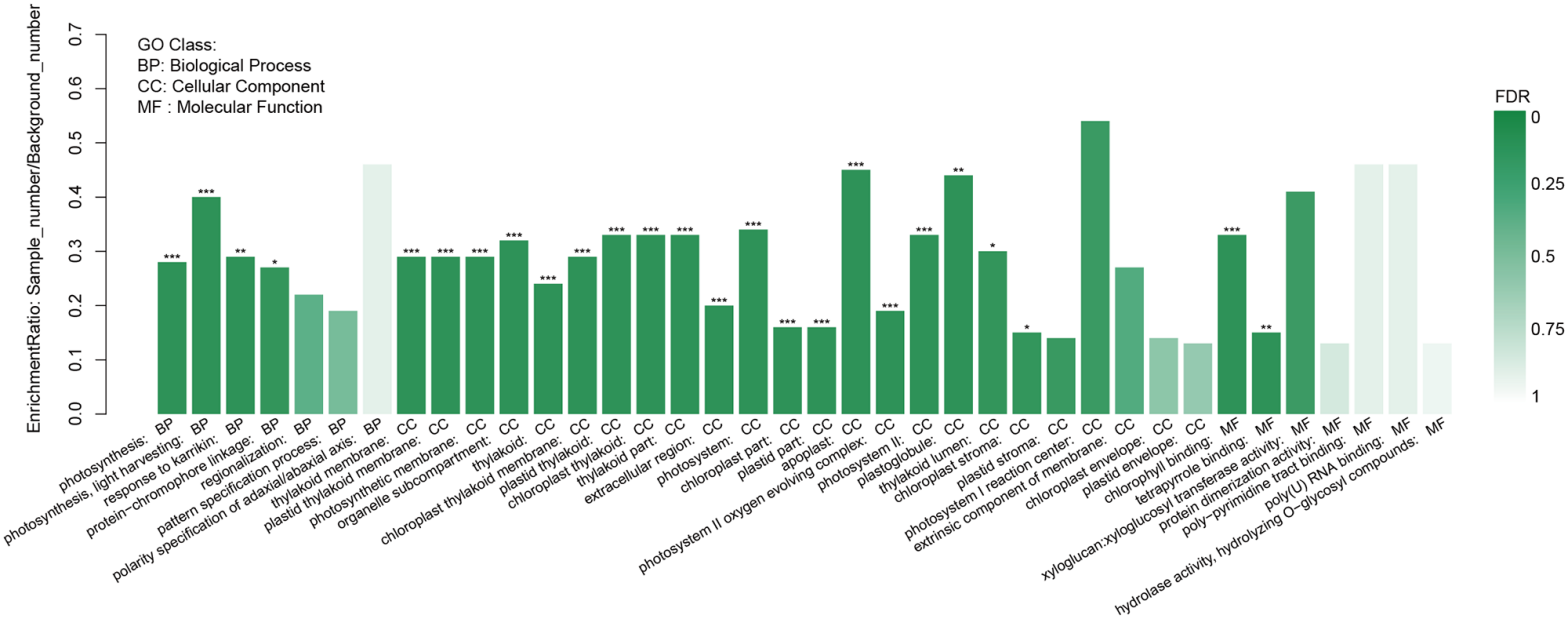
Histogram of GO enrichment distribution of differentially expressed genes (DEG). FDR < 0.001was marked ***, FDR < 0.01 was marked **, FDR < 0.05 was marked *.

The software GOatools was used to further explore the enrichment of DEGs, significance in enrichment was considered if the p-value< 0.05. Fig 7 shows the GO enrichment of DEGs, with the name and classification of GO plotted on the x-axis and the enrichment ration plotted on the y axis. The color indicates the significance of enrichment, whereby increasing color intensity corresponds to greater enrichment of GO with FDR. There were only 25 GO that were significantly enriched in the coGS in contrast with coYP. For example, the enrichment ratio of photosystem II oxygen evolving complex (15/4585), photosynthesis (29/4585), light harvesting (17/4585), chlorophyll binding (18/4585), and photosystem (26/4585) were the highest with an FDR < 0.001; followed by plastoglobule (12/4585), response to karrikin (17/4585) and tetrapyrrole binding (69/4585) with an FDR<0.01. The enrichment ratio of thylakoid lumen (14/4585), protein-chromophore linkage (17/4585), and chloroplast stroma (58/4585) were low with an FDR < 0.05. However, the enrichment ratio of photosystem I reaction center, xyloglucosyl transferase activity, and extrinsic component of membrane were the lowest with FDR ≥ 0.05 (Fig 7, S13 Table).

### KEGG enrichment analysis of DEGs

**Fig 8:**
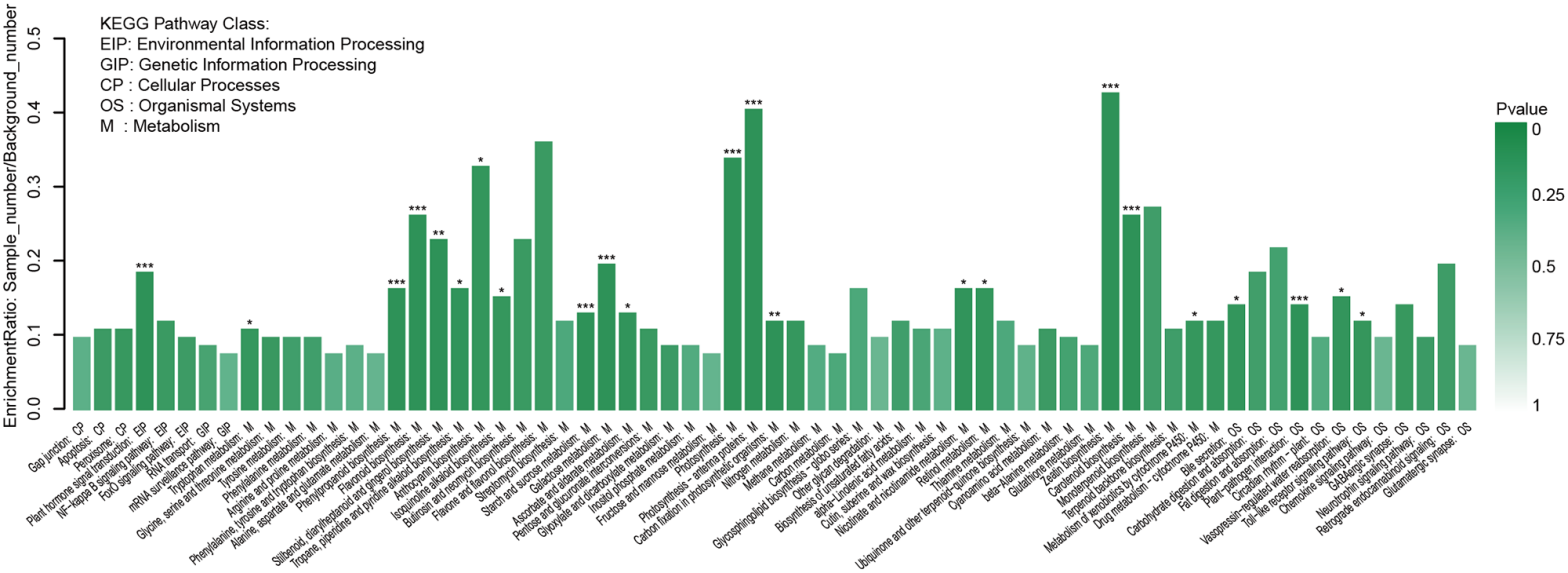
Histogram of KEGG enrichment distribution of DEG. P-value < 0.001was marked ***, P-value < 0.01 was marked **, P-value < 0.05 were marked *.

The DEGs between coYP and coGS were subjected to KEGG pathway enrichment analysis using the software KOBAS. Results showed that 1,670 DEGs were mapped to 226 KEGG pathways (S14 Table). The p-value < 0.05 was set as the threshold of significant enrichment. As a result, 434 (25.99%) DEGs were significantly enriched and were associated with 23 pathways (Fig 8, S14 Table). Among the 23 pathways, 10 pathways indicated the most significant enrichment (p-values < 0.001), which includes zeatin biosynthesis (2.07%, 9/434), photosynthesis-antenna proteins (3.69%, 16/434), photosynthesis (10.37%, 45/434), carotenoid biosynthesis (2.76%, 12/434), and flavonoid biosynthesis (2.76%, 12/434). Moreover, two pathways of carbon fixation in photosynthetic organisms (6.45%, 28/434) and stilbenoid, diarylheptanoid, and gingerol biosynthesis (1.84%, 8/434) were also significantly enriched. Lastly, 11 pathways indicated basic significantly enrichment, including tropane, piperidine, and pyridine alkaloid biosynthesis (1.84%, 8/434), anthocyanin biosynthesis (0.69%, 3/434), toll-like receptor signaling pathway (2.76%,12/434) and retinol metabolism (1.61%, 7/434) (Fig 8, S15 Table). Fig 8 indicates the enrichment ratio of KEGG pathways, where in the intensity of column corresponds to the p-values and enrichment level of KEGG pathways (p-values < 0.001). For example, photosynthesis-antenna proteins was the most significantly enriched, as represented by intense column color.

### Transcription factor forecast and analysis

Transcription factors are proteins that modulate downstream gene expression at different levels by binding to promoter regions of the gene [44, 45]. Furthermore, different transcription factors play a critical role in the regulation of different plant metabolic processes [45]. In this study, 581 transcription factors distributed to 50 transcription factor families were identified in *C*. *officinalis* (S16 Table). Among these, basic Helix-Loop-Helix (bHLH) family (41; 7.06%) was found to be the most abundant, followed by MYB (38; 6.54%), ERF (38; 6.54%), GRAS (34; 5.85%) and bZIP (32; 5.51%) (S16 Table).

## Discussion

As one of the most commonly and extensively used TCM, Corni Fructus has been used to cure liver and kidney diseases, light-headedness, and pain and weakness in the loin and knees for thousands of years. *C*. *officinalis* is a Level 3 protected species, and is one of the 42 national key protected wild species of animals and plants [46]. However, there is little information available with regard to the molecular biology and genomic information of the species, which has impeded the intensive study on its molecular metabolic mechanism and germplasm innovation. In this study, we have performed large-scale transcriptome sequencing of the fruit and leaf tissue of *C*. *officinalis* with the use of advanced high throughput Illumina RNA-seq technology, which allowed the transcriptome of *C*. *officinalis* to be described for the first time and enriched the gene information of *C*. *officinalis*. In addition, the generated transcriptomic data of *C*. *officinalis* can help explore the molecular genetic and biochemical characteristics of *C*. *officinalis* and its related species.

The assembly strategy of Trinity was used in this study as a unified method for transcriptome construction in the absence of a reference genome [28, 47–49]. The N50 is one of the most important indices used to assess the assembly quality, wherein longer N50 length corresponds to higher assembly quality. In our study, the N50 length of unigenes was 1445 bp (Table 1), which showed that the sequence assembly was high in quality and is suitable for intensive research. Moreover, the N50 could be compared with other plant species [49–51]. In addition, the average length of the unigenes was 856 bp (Table 1), which was superior to those in other reported species, such as *Myrica rubra* (531 bp) [52], *Sorbus pohuashanensis* (770 bp) [53] and *Platycladus orientalis* (534 bp) [54]. In this study, a total of 60,971,652 (coGS) and 57954134 (coYP) reads were cleaned and assembled de novo to produce 56,392 unigenes (Table 1). Based on sequence similarity searching, the unigenes were annotated and classified against the NCBI NR database, GO database, KOG terms, and KEGG pathways. Because of the limited genetic information available for *C*. *officinalis*, unknown unigenes were obtained for as many as 5,864 (14.25%) of the sequences (S1 Table). This result has also been found in other species [32]. Possibly, novel genes of *C*. *officinalis* could be found in these unknown unigenes, which were related with some unique biosynthesis processes and pathways in our results. Furthermore, the annotated unigenes of *C*. *officinalis* indicated the highest homology to those of *Vitis vinifera* (18.77%), followed by *Theobroma cacao* (6.17%), and *Coffea canephora* (5.44%) (S1 Table), which may indicate the evolutionary relationship among these species. In spite of a large number of unigene sequences that indicated no matches, many of unigenes were still assigned to a wide range of KOG and GO classifications. The results showed that our transcriptome data included much genetic information on *C*. *officinalis*. These novel unigenes provide an exciting opportunity to study the functional genes in *C*.*officinalis*. Similar findings have been reported in *Chimonanthus praecox* [32], *Panax ginseng* [36] and *Prunus pseudocerasus* [39]. The KEGG function annotation analysis showed that 16,182 unigenes were involved in 371 biosynthesis process. The largest number of unigenes (4,451) was associated with metabolic pathways, followed by those associated with biosynthesis of secondary metabolites. However, the smallest number of unigenes was associated with d-arginine and d-ornithine me tabolism, malaria, taste transduction, asthma, penicillin and cephalosporin biosynthesis, aflatoxin biosynthesis, indole alkaloid biosynthesis, and neuroactive ligand-receptor interaction, which have only 1 matching unigene (S6 Table). All of these data contribute to the study of the metabolic and biosynthesis mechanisms in *C*. *officinalis*.

In addition, we analyzed the DEGs between coGS and coYP using the edgeR software with the set threshold of FDR or *p* value < 0.05. The results showed that many genes indicated significant DEGs between coGS and coYP. We found two photosynthesis-related unigenes (c113745_g1, c95953_g1) that were up-regulated significantly at the coYP tissue and down-regulated at the coGS (S10 Table). In addition, four unigenes (c50578_g2, c138903_g1, c94113_g1, and c32805_g1) associated with chlorophyll a/b binding protein were significantly up-regulated at the coYP tissue and down- regulated at the coGS (S10 Table). In fact, it was well known that the leaf was the only tissue that participates in photosynthesis and chlorophyll biosynthesis. Thus, it was well-understood that the expression of these unigenes is up-regulated in leaf.

Corni Fructus is a popular TCM in China, which came from the fruit of *C*. *officinalis* [46]. Terpeneis is main active ingredients [3]. In the study, we found that the two unigenes c101827_g3 andc78753_g1are related to terpene biosynthesis. The NCBI Blast results also showed that the sequence of c101827_g3 has the greater similarity with *arabidopsis thaliana* terpene synthase 21 (TPS21) (GenBank number: NM_122301) [55], which is the main enzyme in the biosynthesis of terpeneis in *arabidopsis thaliana*. As we know, the fruit of *C*. *officinalis* is its medicinal parts, and the terpene composition should mainly exist in the fruit of *C*. *officinalis*. In our study, the unigenes just were significantly up-regulated in coGS tissue and down-regulated in coYP (S10 Table). The results hinted that the terpene is mainly found in the fruit of *C*. *officinalis*, which is consistent with earlier reports [3, 5].

As everyone knows, MVA (mevalonate) and EMP (Embden-Meyerhof-Parnas pathway) are the basic pathways in the biosynthesis and emission of terpenes, which have been explored in many species [56–59]. In this study, we found many unigenes involved in terpene biosynthesis based on the unigenes functional annotation. In *C*. *officinalis*, terpenoid biosynthesis enzymes involved in the MVA and MEP pathways were distinguished with TPS (trehalose-phosphate synthase, c79346_g1, c100180_g2, c151387_g1, c115293_g1, c151414_g1), DXS (1-deoxy-D-xylulose-5-phosphate synthase, c19035_g1, c87460_g2,c93144_g1), and DXR (1-deoxy-D-xylulose 5-phosphate reductoisomerase, c7855_g1, c112578_g1, c147202_ g1) (S3 Fig). All these enzymes play a pivotal role in terpene biosynthesis, which proved the involvement of terpene metabolic pathways in *C*. *officinalis*. Furthermore, there were multiple unigene sequences annotated to the same enzyme, in which unique sequences indicated different fragments of a single unigene, different members of a gene family, or both, this finding was similar to a previous report in American ginseng [59].

## Conclusions

This was the first comprehensive report on *C*. *officinalis* transcriptome. The study presents the transcriptome sequencing results and analysis of *C*. *officinalis* leaf and fruit using the Illumina transcriptome sequence platform. A total of 56,392 unigenes with 48,264,743 bp were generated. In addition, we have explored DEGs between the leaf and fruit of *C*. *officinalis* using the edgeR database. This study has enriched the genetic data of *C*. *officinalis* and the indicated the potential of transcriptome sequencing for functional gene research in species where genomic sequence data are not yet available. We are confident that the results will lay the foundation for further functional genomics and molecular and molecular metabolic mechanism studies on *C*. *officinalis*.

## Supporting information

S1 Table Statistical table of alignment species of unigenes with NR database. (XLS)

S2 Table Summary of GO term classification for the *C*. *officinalis* transcriptome. (XLS)

S3 Table Summary of GO term level3 classification for the *C*. *officinalis* transcriptome. (XLS)

S4 Table Summary of GO term level4 classification for the *C*. *officinalis* transcriptome. (XLS)

S5 Table Summary of KOG functional classificationfor the *C*. *officinalis* unigenes. (XLS)

S6 Table Summary of KEGG pathways involved in the *C*. *officinalis* transcriptome. (XLS)

S7 Table Five branches of KEGG pathways of the *C*. *officinalis* transcriptome. (XLS)

S8 Table The number of reads supported unigenes of coYP and coGS. (XLS)

S9 Table FPKM expression quantity of every gene in coGS and coYP. (XLS)

S10 Table Summary of significance DEGs coYP versus coGS. (XLS)

S11 Table Summary all of DEGs coYP versus coGS. (XLS)

S12 Table Summary of the significantly DEGs in GO classification in coYP versus coGS. (XLS)

S13 Table Summary of GO enrichment of significant DEGs with coYP versus coGS. (XLS)

S14 Table Summary of KEGG pathway mapped DEGs with coYP versus coGS. (XLS)

S15 Table Summary of KEGG pathway enrichment of significant DEGs with coYP versus coGS. (XLS)

S16 Table Summary of transcription factors in *C*. *officinalis* transcriptome. (XLS)

S1 Fig Distribution of base composition, quality and mean error. (A) distribution of base composition of coGS. (B) distribution of base composition of coYP. (C) distribution of base qualities of coGS. (D) distribution of base qualities of coYP. (E) distribution of base mean error of coGS. (F) distribution of base mean error of coYP.
(TIF)

S2 Fig Distribution of gene FPKM against unigenes of coYP and coGS. (TIF)

S3 Fig Possible iridoid synthetic pathways of *C*. *officinalis*. (TIF)
