# Supplementary figures and images for: De novo transcriptomic analysis of leaf and fruit tissue of Cornus officinalis using Illumina platform
Source: PLoS One. 2018 Feb 16;13(2):e0192610. doi: 10.1371/journal.pone.0192610 (PMC5815590; doi:10.1371/journal.pone.0192610)

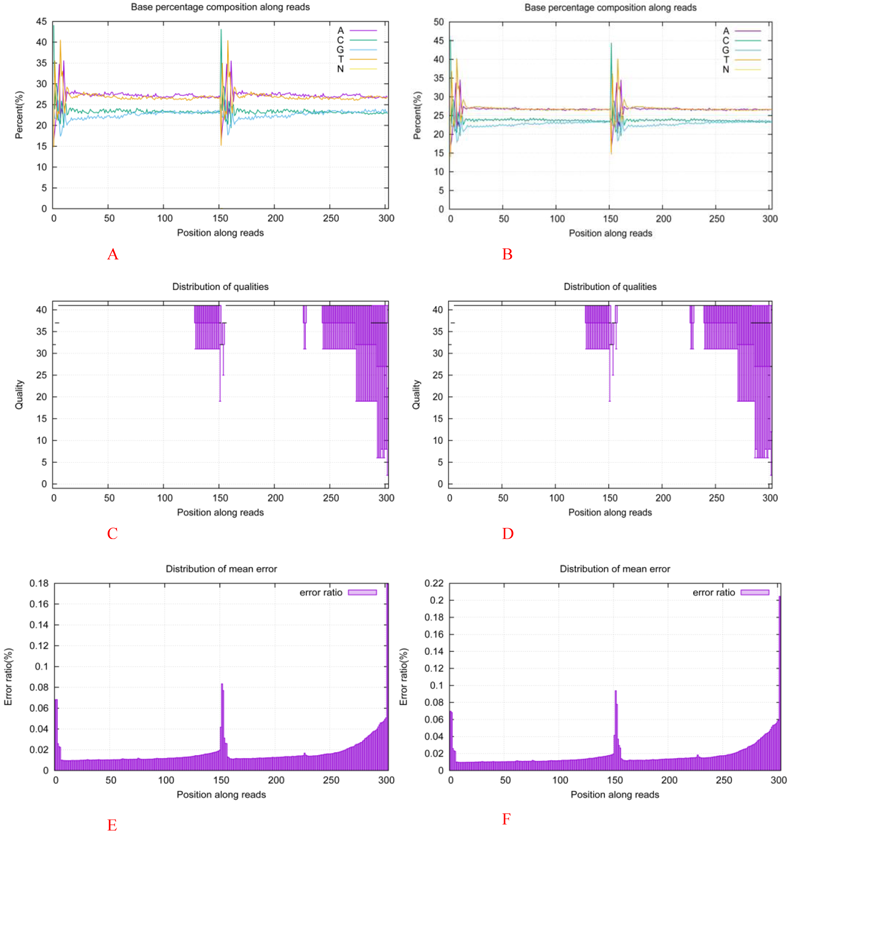

Supplement: S1 Fig — (A) distribution of base composition of coGS. (B) distribution of base composition of coYP. (C) distribution of base qualities of coGS. (D) distribution of base qualities of coYP. (E) distribution of base mean error of coGS. (F) distribution of base mean error of coYP. (TIF) [file pone.0192610.s017.tif]

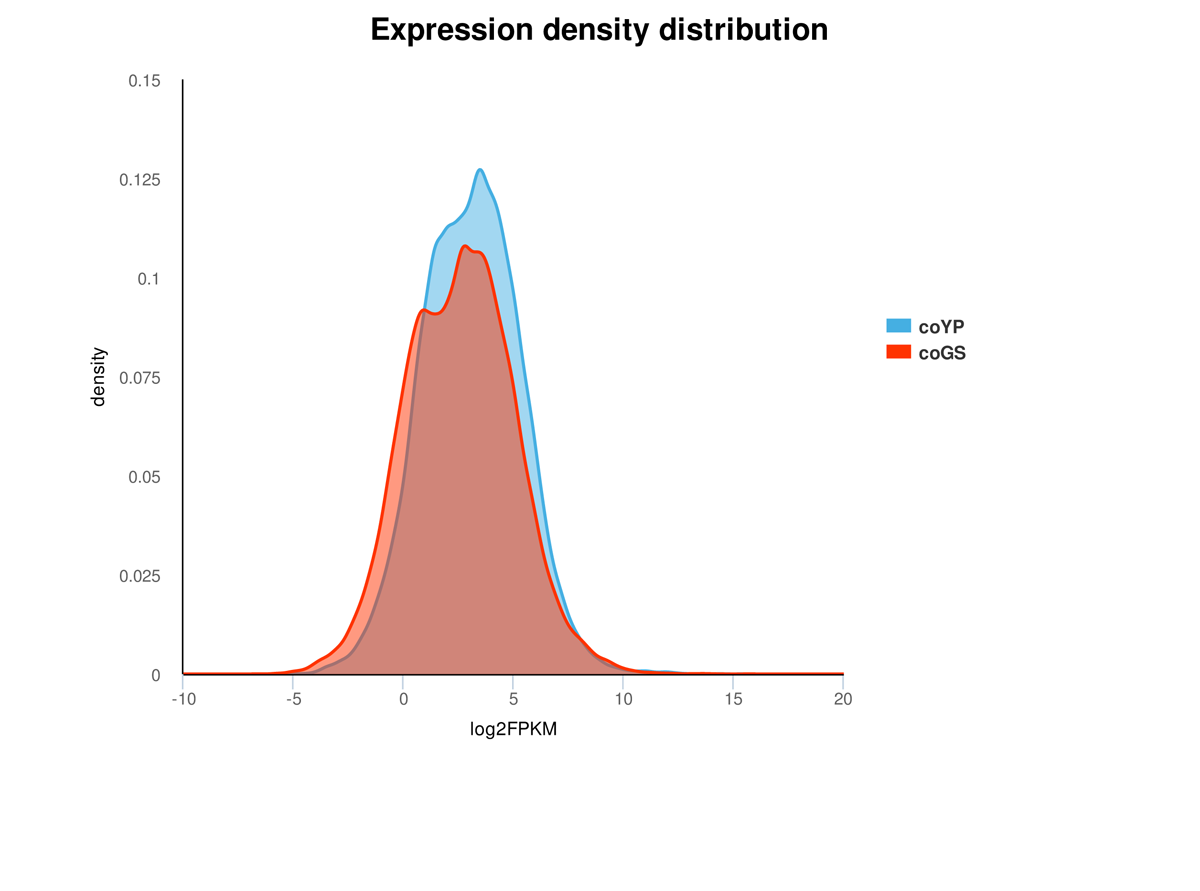

Supplement: S2 Fig — (TIF) [file pone.0192610.s018.tif]

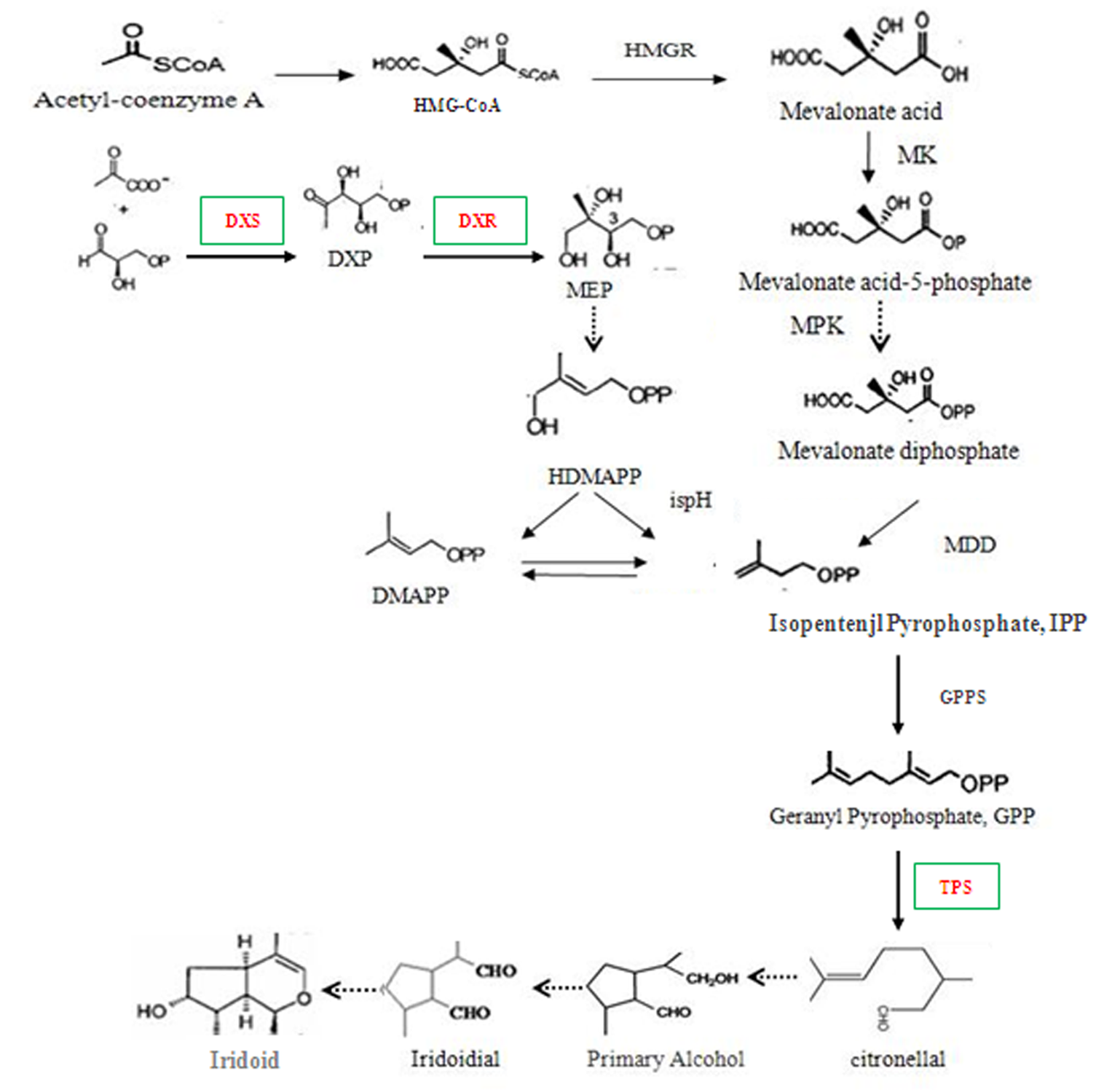

Supplement: S3 Fig — (TIF) [file pone.0192610.s019.tif]
